# Persisting sicca complaints in sarcoidosis

**DOI:** 10.3389/fmed.2022.975122

**Published:** 2022-08-31

**Authors:** Benedikt Hofauer, Miriam Wiesner, Zhaojun Zhu, Konrad Stock, Friedhelm Peltz, Klaus Thuermel, Ulrich Strassen, Felix Johnson, Andreas Knopf

**Affiliations:** ^1^Department of Otorhinolaryngology/Head and Neck Surgery, Klinikum Rechts der Isar, Technical University of Munich, Munich, Germany; ^2^Department of Nephrology, Klinikum Rechts der Isar, Technical University of Munich, Munich, Germany; ^3^Department Pneumology, Klinikum Rechts der Isar, Technical University of Munich, Munich, Germany; ^4^Department Nephrology and Rheumatology, Klinikum Augustinum, Munich, Germany; ^5^Department of Otorhinolaryngology/Head and Neck Surgery, University Medical Center Freiburg, University of Freiburg, Freiburg, Germany

**Keywords:** sarcoidosis, head and neck, salivary glands, parotid gland, submandibular gland, sonography, elastography

## Abstract

**Background:**

Sarcoidosis is a systemic inflammatory disease that is characterized by non-caseating granulomas. Besides the lung as classical site of involvement, extrapulmonary manifestations are common, for example cervical lymph nodes or the salivary glands. The aim of this investigation is the analysis of the long-term course of glandular symptoms with a focus on persisting sicca symptoms.

**Materials and methods:**

All patients with the diagnosis of sarcoidosis over a period of 20 years in the departments of otorhinolaryngology, nephrology and pneumology were identified. In addition to clinical examinations and functional evaluation of the salivary glands, a sonographic examination of the salivary glands was carried out.

**Results:**

A total of 76 patients were included in the study (age 35.1 ± 21.6 years). At baseline, 32 out of 76 patients were suffering from xerostomia, 36 from dry eyes. While other salivary gland symptoms, such as gland enlargement, pain or facial nerve impairment, dissolved during the further course of the disease, xerostomia was still present in 29 and dry eyes in 35 out of 76 patients at the time of follow-up (which took place on average after 88.2 months).

**Conclusion:**

Sicca symptoms persist in patients with the diagnosis of sarcoidosis, while other salivary gland symptoms completely dissolve during the further course of the disease. This development appears to be independent of the type of therapy and should be considered during the follow-up of these patients, since sicca symptoms can cause further ocular, oral and dental damage.

## Introduction

Sarcoidosis is an inflammatory multisystem granulomatous disease of unclear origin that affects individuals worldwide and is pathologically characterized by non-caseating granulomas (1–3). It predominantly affects younger adults and typically manifests itself as bilateral hilar adenopathy and/or pulmonary reticular opacities—however, up to 30% of patients present with extrapulmonary sarcoidosis (4, 5). Sarcoidosis can ultimately affect all organ systems with different degrees of severity, while the most common sites of extrapulmonary disease are the skin, joints, eyes, reticuloendothelial system, musculoskeletal system, exocrine glands, heart, kidney and central nervous system. In approximately eight percent of patients, extrapulmonary manifestations are the only manifestation of the disease (6–8).

Sarcoidosis can also affect the salivary and lacrimal glands—typical signs for an involvement of these glands are xerostomia, dry eyes or gland enlargement, which is similar to the clinical manifestations of Sjögren's syndrome or IgG4-related systemic disease (9, 10). While on the one hand the clinical presentation, especially of sarcoidosis and Sjögren's syndrome, might be similar (or can also occur concomitantly), there are on the other hand clear differences—among other things Sjögren's syndrome is a chronic disease with a typical sonographic presentation within the salivary gland in 50–60% of patients, whereas sarcoidosis is a self-limitating disease without typical sonographic changes within affected salivary glands (11–14).

Due to the similar clinical symptoms, a previously known sarcoidosis is an exclusion criterion in various established classification criteria for Sjögren's syndrome (15). However, it is not specified in more detail to what extent the time difference between diagnosis of the sarcoidosis and the time of the suspected Sjögren's syndrome play a role and whether a past and healed sarcoid disease may enable the diagnosis of a Sjögren's syndrome that occurs later. A possible explanation, why this was not taken into account when creating the mentioned classification criteria, could be the lack of data on the long-term course of sarcoidosis affecting the salivary and lacrimal glands. No data are available so far, particularly on the course of dry eyes and mouth. In the case of these complaints, it would be important to know how long patients require follow-up, so that preventive measures can be initiated early enough to counteract or identify early any subsequent problems, such as damage to the cornea in the eye area or caries of the teeth.

For this reason, the aim of this study was to examine the long-term clinical symptoms in patients with a diagnosis of sarcoidosis with a special focus on symptoms originating from the salivary and lacrimal glands.

## Materials and methods

### Study population

Patients with the diagnosis of sarcoidosis and treated either at the department of otorhinolaryngology/head and neck surgery, department of pneumology or department of nephrology at the Klinikum rechts der Isar, Technical University of Munich, Germany, between 01.01.2000 and 31.12.2018 were identified and offered to take part in this study. Sarcoidosis was diagnosed according the American Thoracic Society/European Respiratory Society/World Association of Sarcoidosis and Other Granulomatous Disorders (WASOG) 1999 statement on sarcoidosis. All examinations were performed from October 2017 to February 2018. The study protocol was in accordance with the Declaration of Helsinki. The Institutional Review Board of the Medical Faculty, Technical University of Munich, reviewed and approved the protocol (468/18). Written informed consent was obtained from all participants prior to the begin of the examination.

### Clinical parameter

Relevant symptoms (e.g., xerostomia, dry eyes, parotid gland enlargement, facial nerve affection, uveitis) were evaluated with visual analog scales at baseline and at the time of follow-up (with a range from 0 to 10). Level of initial ACE (angiotensin converting enzyme) and sIL2-R (soluble interleukin 2-receptor) were collected. Unstimulated salivary flow (UWSF) and Schirmer-test were measured to evaluate salivary and lacrimal gland function. Chest X-ray has been scored according to the Scadding stages (16, 17).

### Sonographic evaluation

All sonographic examinations were performed simultaneously with the other diagnostic evaluations (Acuson S2000, 9L4, Siemens Healthcare, Erlangen, Germany). B-mode sonography was performed on both parotid and submandibular glands. The echostructure of the parotid and submandibular glands in B-mode sonography was graded on a scale of 0–4 according to a previously published scoring system: grade 0: normal, homogeneous gland; grade 1: mild parenchymal inhomogeneity (PIH), hypoechoic areas <2 mm; grade 2: evident PIH, hypoechoic areas of 2–6 mm; grade 3: gross PIH, hypoechoic areas >6 mm; grade 4: adipose degeneration of the gland, adipose tissue echogenicity and parenchymal atrophy (11, 18). The B-mode result was scored as abnormal if the score was 2 or higher, which had proved to be the optimal cut off in previous studies. Sonographic images representing the average echogenicity of the examined salivary gland were archived.

### Statistical analysis

Statistical analysis was done using version 28.0 of the Statistical Package for Social Sciences software (SPSS, Chicago, IL, USA). Descriptive data are reported as mean ± standard deviation, if not otherwise stated. Normal distribution of variables was tested by using the Kolmogorov-Smirnov test. Paired *t*-tests were used for normally distributed variables and Wilcoxon test for not normally distributed variables. Pearson correlation coefficient was used for analysis of correlations (*r*: 0.8–1.0: very strong correlation, *r*: 0.6–0.79: strong correlation, *r*: 0.40–0.59: moderate correlation, *r*: 0.20–0.39: weak correlation, *r*: 0.00–0.19: very weak correlation). *p*-values of <0.05 were considered as statistically significant.

## Results

### Study population

Of the patients who were identified with a diagnosis of sarcoidosis, 76 could be included in the further investigations. Baseline details on the study population are illustrated in Table 1. In the vast majority of cases (72/76, 95%), diagnosis was based on histological samples. Biopsies were taken from the lymph nodes (32/76), lung (28/76), skin (14/76), salivary glands (6/76) and other locations (9/76, e.g., liver, kidney, pharynx, stomach), with biopsies taken from multiple sites in some patients.

**Table 1:** Baseline details on the study population (n = 76).

Gender distribution (% female)	65.8
Age at diagnosis (years)	35.1 ± 21.6
**Treating department (** ***n*** **/%)**	
ENT	23/30.3
Rheumatology	27/35.5
ENT + Rheumatology	15/19.7
Pneumology	11/14.5
ACE (U/l)	85.3 ± 59.1
sIL-2R (U/ml)	1461.3 ± 959.4
**Chest X-Ray (** ***n*** **/%)**	
Normal	6/7.9
Bihilar lymphadenopathy (LAP)	26/34.2
Bihilar LAP with lung involvement	27/35.5
Lung involvement without bihilar LAP	4/5.3
Lung fibrosis	0/0
ENT symptoms (***n***/%)	60/78.9

### Baseline clinical parameter depending on salivary gland involvement

At baseline, 32 out of 76 patients were suffering from xerostomia and patients graded their complaints with a mean of 2.61 ± 3.49. Thirty-six patients were suffering from dry eyes, on the visual analog scales the patients graded the extent with 2.74 ± 3.34. Other salivary gland and related symptoms at baseline are illustrated in Table 2. Xerostomia at baseline correlated with other salivary gland complaints, such as gland enlargement (*r* = 0.349, *p* = 0.002) and pain within the glands (*r* = 0.410, *p* < 0.001). Patients with xerostomia also complained about dry eyes more often (*r* = 0.472, *p* < 0.001). Subjective and objective salivary gland impairment did not correlate with any sonographic abnormalities.

**Table 2:** Comparison between salivary gland and related symptoms at baseline and during follow-up.

	**Baseline**	**Follow-up**	**p-value**
Xerostomia	32/76	29/76	0.681
Salivary gland enlargement	18/76	7/76	0.005
Salivary gland pain	8/76	3/76	0.059
Facial nerve impairment	13/76	1/76	<0.001
Fever	16/76	1/76	<0.001
Uveitis	13/76	5/76	0.005

### Clinical parameter at follow-up

**Figure 1:**
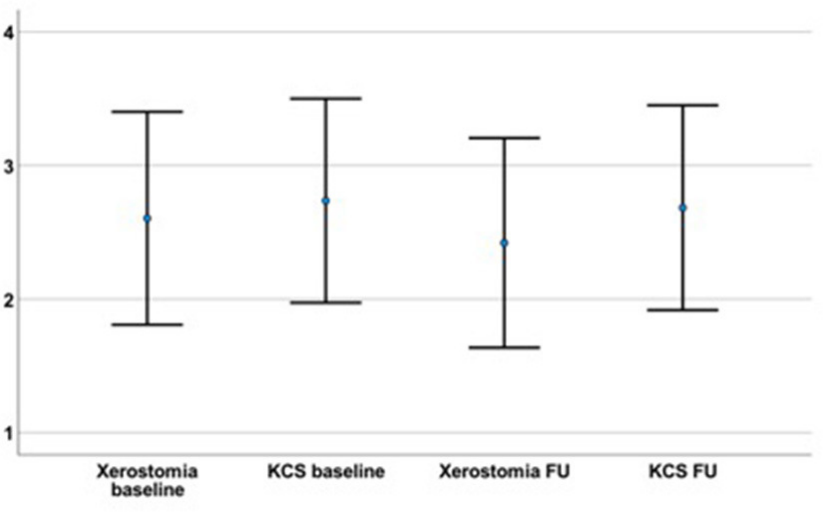
The severity of the initial xerostomia (2.61 ± 3.49) and dry eyes (KCS; 2.74 ± 3.34) had not declined at the time of the follow-up (FU; time interval between initial diagnosis and FU on average 88.2 ± 84.0 months; xerostomia = 2.42 ± 3.43; *p* = 0.681; KCS = 2.68 ± 3.36; *p* = 0.861).

**Figure 2:**
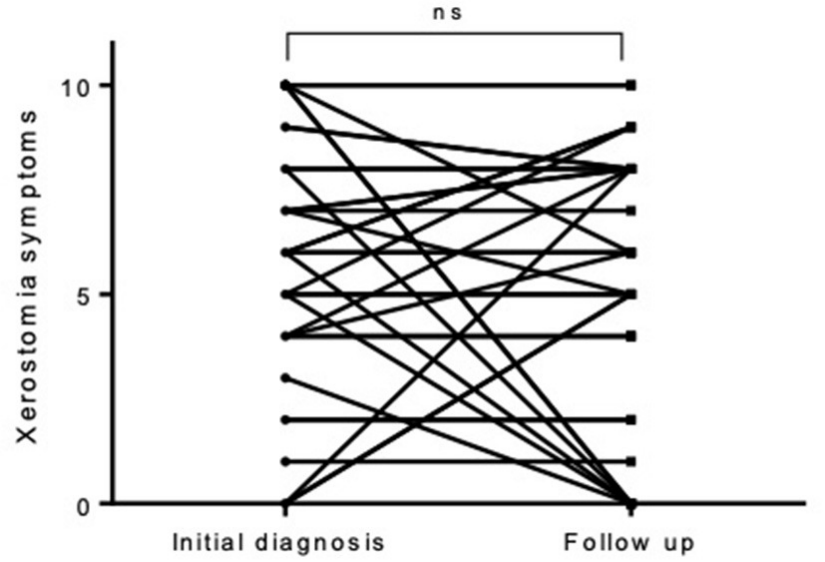
In individual cases, there was a complete reduction of the initially complained xerostomia—in most cases, however, it did not change during the observation period.

The follow-up took place on average after 88.2 months (± 84.0) after the initial diagnosis (median 61.0 month, minimum 9 months, maximum 442 months). At this time, 29 out of 76 included patients were suffering from xerostomia and the extent was graded with a mean value of 2.42 ± 3.43. Dry eyes were present in 35 out of 76 patients during the follow-up with an extent of 2.68 ± 3.36 (Figures 1, 2). The unstimulated whole salivary flow (UWSF) was 1.21 ml/5 min (±1.04) and the tear flow, evaluated by the Schirmer-test, was 16.88 mm/5 min (±10.22). In patients suffering from xerostomia during the follow-up, the UWSF was 0.92 ml/5 min (±0.97), compared to 1.41 ml/5 min (±1.04) in patients without xerostomia (*p* = 0.047). In patients suffering from dry eyes during the follow-up, the Schirmer-test was 14.99 mm/5 min (±10.48), compared to 18.47 mm/5 min (±9.82) in patients without dry eyes (*p* = 0.151). Other salivary gland and related symptoms at baseline are illustrated in Table 2. The time interval between initial diagnosis and follow-up did not correlate with remaining clinical symptoms. There were no significant differences between patients with and without improvement of xerostomia regarding gender (*p* = 0.953), treatment with steroids or other medications (*p* = 0.117 and *p* = 0.511), age at initial diagnosis (*p* = 0.822) or time interval between initial diagnosis and follow-up (*p* = 0.923).

Out of the 76 patients, 58 received an immunosuppressive therapy, mainly steroids. The evaluation showed that there was no difference between the patients with and those without medical therapy with regard to the complained xerostomia and dry eyes. Also, with regard to objective parameter, the saliva and tear production, there was no difference between patients treated with or without additional immunosuppression (*p* = 0.460 and *p* = 0.318, respectively).

B-mode sonography of the salivary glands resulted in mainly normal scores within the applied scoring systems. The mean score for the right (0.58 ± 0.57) and left submandibular gland (0.56 ± 0.68), as well as the right (0.32 ± 0.72) and left parotid gland (0.41 ± 0.73), were within normal limits. There were no statistically significant differences in sonographic findings between patients with initial a persistent xerostomia.

## Discussion

The current study evaluated the long-term clinical symptoms in patients with a diagnosis of sarcoidosis with a special focus on symptoms originating from the salivary and lacrimal glands. This should provide first information on how long a former sarcoidosis still has to be considered as a differential diagnosis for certain symptoms.

The focus of our clinical follow-up was on symptoms that emanate from a manifestation of the disease in the area of the salivary and lacrimal glands. It was shown here that acute symptoms of salivary gland involvement, such as swelling or pain, did not persist for a long period of time, while dry mouth (and also dry eyes) can persist over a period of years without any significant dynamics. It could also be shown that xerostomia and dry eyes are the most common salivary gland-related symptoms at the time of initial diagnosis but also in the further course of the disease and that approximately half of all patients are affected (19). This is an important information for the treating physicians, since patients may not receive long-term follow-up care because the leading symptoms have subsided, but xerostomia and dry eyes persist over a long period of time and also do not appear to be influenced by different medical treatments. However, continued care of these patients in particular, similar to that for patients with Sjögren's syndrome, by ophthalmologists and oral specialists, makes sense so that subsequent problems can be avoided (in this manuscript Sjögren's syndrome is used in the sense of a diagnosed disease, not synonymous with sicca symptoms). Xerostomia can cause difficulty in feeding or sustained speech, and problems wearing dentures in elderly patients. Injuries to the oral mucosa with sometimes significantly protracted healing processes are also observed (20, 21). Persistent dry eyes will lead to chronic irritation and structural damage such as corneal ulceration (22). The large number of complications to be expected from untreated sicca symptoms makes it clear why care for these patients is necessary. While the entire cohort rated xerostomia with a score of 2.42, which would represent a low burden, the average xerostomia score among patients who complained of dry mouth at all was 6.34 ± 2.41, which is significantly higher. In a patient reported index for Sjögren's syndrome, the so called ESSPRI (a combination of a visual analog scale of three symptoms—pain, fatigue and dryness), an acceptable symptom state is defined as a score of 5.0 or less (23, 24). No comparable scoring system is available for sarcoidosis containing the evaluation of xerostomia, but this comparison illustrates the severity of the persisting xerostomia in the presented cohort.

EULAR recently published a recommendation for the symptomatic treatment of sicca symptoms in patients with Sjögren's syndrome, which could also provide good guidance for patients with sarcoidosis (25). However, it should be noted that the measurement results of the Schirmer-test were within normal values—when measuring the unstimulated salivary flow rate actually a reduced value was shown, which was still above the limit of 0.1 ml/min, which is used as a cut-off in suspected Sjögren's syndrome.

There are hardly any studies on dry mouth and eyes in patients with sarcoidosis, making comparisons with other studies or populations difficult. One study by Mansour et al. on the clinical and salivary evaluation of sarcoidosis and Sjögren's syndrome observed dry mouth symptoms in 58.3% of all included patient with sarcoidosis and dry eyes symptoms in 33.3% of the patients. This study also lists other oral symptoms, such as difficulties in swallowing (16.7%) or changes in taste (25%), and other ocular symptoms, such as itching eyes (33.3%) or intolerance to light (16.7%). Different to our study, the stimulated salivary flow was measured. With a value of 0.41 ml/min it was below the normal value of 0.7 ml/min (19). We also evaluated the percentage of patients with sicca symptoms within patients, who were treated primarily in the department of otorhinolaryngology compared to all the other departments, and did not observe a different distribution, neither at baseline, nor at follow-up. It is not clear yet, why sarcoidosis causes this loss of salivary gland function—potential explanations are either that local inflammation induces a fall in acinar function and/or loss of salivary duct patency.

The multimodal follow-up was carried out in all patients at different times in relation to the initial diagnosis and in some cases, examinations were carried out during the follow-up that were not carried out as part of the initial diagnosis and therefore no statement can be made about the development over time. This represents a limitation of this study.

The work presented is part of a larger project in the field of sarcoidosis, which has already resulted in another publication on the multimodal sonographic assessment of salivary gland alterations in sarcoidosis (26). Both works are based on an evaluation of the same patient population, but analyze different aspects of the disease. With this work, we present new insights into the long-term course (and long-term persistence) of sicca symptoms in patients with sarcoidosis, for which there was previously no reported evidence.

In summary, it can be said that the subjectively felt dry mouth and eyes in patients with sarcoidosis persist over a long observation period, while other gland-related symptoms completely regress. This course appears to be independent of the type of therapy. It cannot be said with certainty whether this is due to persistent damage to the glandular parenchyma, since these appear normal or without characteristic changes, at least on sonography. In view of the knowledge about these persistent symptoms, however, it seems advisable that patients with a xerostomia in particular are given long-term follow-up care so that possible consecutive complications can be avoided. This study provides an unprecedented insight into the long-term symptoms related to the large head and neck glands and is therefore also of clinical relevance, especially with regard to the differentiation from other diseases such as Sjögren's syndrome.

## Transparency statement

The work presented is part of a larger project in the field of sarcoidosis, which has already resulted in another publication on the multimodal sonographic assessment of salivary gland alterations in sarcoidosis (26). Both works are based on an evaluation of the same patient population, but analyze different aspects of the disease.

## Data availability statement

The raw data supporting the conclusions of this article will be made available by the authors, without undue reservation.

## Ethics statement

The studies involving human participants were reviewed and approved by Institutional Review Board of the Medical Faculty, Technical University of Munich (468/18). The patients/participants provided their written informed consent to participate in this study.

## Author contributions

BH, MW, ZZ, KS, FP, KT, US, FJ, and AK contributed substantially to the conception or design of the work, the acquisition and analysis of data for the work. All authors were involved in drafting the work or revising it critically for important intellectual content and provided approval for publication of the content. All authors agreed to be accountable for all aspects of the work in ensuring that questions related to the accuracy or integrity of any part of the work are appropriately investigated and resolved.

## Conflict of interest

The authors declare that the research was conducted in the absence of any commercial or financial relationships that could be construed as a potential conflict of interest.

## Publisher's note

All claims expressed in this article are solely those of the authors and do not necessarily represent those of their affiliated organizations, or those of the publisher, the editors and the reviewers. Any product that may be evaluated in this article, or claim that may be made by its manufacturer, is not guaranteed or endorsed by the publisher.
